# Supplementary material for: Integrating Proteomics and Transcriptomics Reveals the Potential Pathways of Hippocampal Neuron Apoptosis in Dravet Syndrome Model Mice
Source: Int J Mol Sci. 2024 Apr 18;25(8):4457. doi: 10.3390/ijms25084457 (PMC11050081; doi:10.3390/ijms25084457)
Supplement: Supplementary file 1 [file ijms-25-04457-s001.zip › supplementary methods S1.pdf]

Proteomics Methods and Procedures

1.Protein extraction

1.1. Remove frozen samples, add liquid nitrogen and grind thoroughly, weigh the appropriate amount of sample and transfer to a 1.5mL centrifuge tube.

1.2. Add SDS lysis solution(1% SDS, 50mM Tris, PH8.1, sodium pyrophosphate, β-glycerophosphate, EDTA)(Beyotime, Shanghai) , add phosphatase inhibitor(Roche, China) and protease inhibitor(Amresco, USA) to a final concentration of 1 mM.(The main components of SDS lysate were 50 mM Tris (PH8.1), 1% SDS, and various inhibitors such as sodium pyrophosphate, β-glycerophosphate, sodium fluoride, EDTA, leupeptin, etc., which could effectively inhibit protein degradation.)

1.3. Ultrasonic breakage on ice, power 80W, ultrasonic 1.0s, off 1.0s, total 2min, repeat twice.

1.4. centrifuge the solution at 12000rpm for 10min at 4°C, take the supernatant, and centrifuge again to take the supernatant

1.5. The supernatant is the total protein solution of the sample, and the protein concentration is determined and stored at -80°C after dispensing.

The concentration of the sample was determined using the BCA protein concentration assay(Thermo Scientific, USA).

2. Experimental procedure of the BCA method

2.1. Configure the required volume of color development solution according to the BCA kit instructions(Thermo Scientific, USA), based on buffer A: Buffer B = 50:1 (v/v).

2.2. Remove a portion of the protein solution to be tested and dilute it with ultrapure water (to prevent the concentration from being too high and outside the working range of the standard curve).

2.3. Prepare a clean 96-well plate by adding the following gradient of BSA standard protein solution: 0,1,2,4,8,12,16,20 μL, and then add the corresponding volume of ultrapure water to each well to supplement the volume to 20 μL.

2.4. Add 2 μL of the protein solution to be tested into the 96-well plate, set up three replicate wells for each sample, and similarly replenish the volume to 20 μL.

2.5. Add 200μL of pre-configured color development solution into each well (color development solution must be used now!). The reaction was carried out at 37°C for 30min.

2.6. Determine the absorbance value (wavelength 562 nm) using an enzyme marker.

2.7. Calculate the standard curve based on the known concentration and absorbance value of the

standard protein solution, and substitute the absorbance value of the sample to be tested to calculate the protein concentration value.

3.SDS-polyacrylamide gel electrophoresis

3.1. 10 μg of protein was taken from each sample and separated by 12% SDS-PAGE;

3.2. The separated gel was stained with Caulmers Brilliant Blue using eStain LG Protein Stainer(GenScript Biotechnology Co., Ltd,Nan Jing).

3.3 The stained gel was imaged using a fully automated digital gel image analysis system.(Tanon, Shanghai).

4 . Trypsin digestion

4.1. According to the determined protein concentration, take 50 μg of protein for each sample, and dilute and adjust the different groups of samples to the same concentration and volume with lysis solution.

4.2. Add Dithiothreitol(DTT,Titan,Shanghai) to the above protein solution so that the final concentration of DTT is 5 mM, mix well and incubate at 55°C for 30 min.

4.3. Cool on ice until it reaches room temperature. (Note: the solution should not be too cold or too hot when felt by hand.)

4.4. Add the appropriate volume of iodoacetamide(IAA,Sangon, Shanghai) to give a final concentration of 10 mM, mix thoroughly and leave at room temperature for 15 min, protected from light.

4.5. Add 6 times the volume of acetone(IAA,Sangon, Shanghai) to the above solution to precipitate the protein and leave at -20°C for more than four hours or overnight.

4.6. Collect the precipitate by centrifugation at 8000×g for 10 min at 4°C and evaporate the acetone for 2-3 min.

4.7. Add 100 μL of TEAB (200 mM)(Sangon, Shanghai) to re-dissolve the precipitate, add 1 mg/ml Trypsin-TPCK(Sangon, Shanghai) to 1/50th of the sample mass, and digest overnight at 37°C.

4.8. Lyophilize the digested samples and store at -80°C.

5. Peptide labeling

5.1. Add 50 μL of 100 mM TEAB buffer to the lyophilized sample, vortex to mix, and perform the labeling reaction in a 1.5 mL Ep tube.

5.2. Remove the TMT reagent(Thermo Fisher, USA) from the refrigerator, equilibrate to room

temperature, add 88 μL of anhydrous acetonitrile, vortex for 5 min, and centrifuge.

5.3. Take 41 μL of TMT reagent and add it to the sample, vortex to mix well and leave it at room temperature for 1 h. 4.

5.4. Add 8 μL of 5% hydroxylamine to terminate the reaction for 15 min, lyophilized, and stored at -80°C.

6. Separation by reversed-phase chromatography

Liquid Chromatography: Agilent 1100 HPLC.(high pH separation liquid chromatograph, Aglient, China)

Column: Agilent Zorbax Extend - C18 narrow column, 2.1×150 mm, 5 μm

Detection wavelength: UV 210 nm

Mobile phase A: ACN-H2O (2:98, v/v), pH adjusted to 10 with ammonia.(Acetonitrile,ACN, Thermo Fisher, USA)

Mobile phase B: ACN-H2O (90:10, v/v), pH adjusted to 10 with ammonia.

Flow rate: 300 μL/min;

Gradient elution conditions: 0~8 min, 98% A; 8~8.01 min, 98%~95% A; 8.01~48 min, 95%~75% A; 48~60 min, 75~60% A; 60~60.01 min, 60~10% A; 60.01~70 min, 10% A; 70~70.01 min, 10~98% A; 70.01 to 75 min, 98% A.

The samples were collected from 8-60 min, and the eluent was collected into centrifuge tubes 1-15 at one-minute intervals in sequence, and then the samples were collected repeatedly according to 1→15. After collection, vacuum freeze-drying and evacuation, the samples were frozen and stored for mass spectrometry.

7. Chromatography and Mass Spectrometry

<Chromatographic conditions >

All analyse were performed by a Q-Exactive mass spectrometer (Thermo Fisher, USA,) equipped with a Nanospray.

The samples were at a flow rate of 2 μl/min onto a pre-column ,Acclaim PepMap100 100 μm×2cm (RP-C18, Thermo Fisher, USA)(loading time 3 min) , and then they were separated at a flow rate of 300nL/min onto an analytical column, Acclaim PepMap RSLC, 75 μm×50cm (RP-C18, Thermo Fisher)

Mobile phase A: H2O-FA (99.9:0.1, v/v);

Mobile phase B phase: ACN-H2O-FA (80:19.9:0.1, v/v/v);

Gradient elution conditions: 0-40 min, 5-28% B; 40-60 min, 28-42% B; 60-65 min, 42-90% B; 65-75 min, 90% B.

<Mass Spectrometry Conditions>

The primary MS mass resolution was set to 60000, the automatic gain control value was set to 1e6, and the maximum injection time was 50 ms; the mass spectral scan was set to a full-scan charge-to-mass ratio m/z range of 350-1500; all MS/MS plot acquisitions were accomplished using high-energy collisional cleavage in the data-dependent positive-ion mode, with the collisional energy set to 36; the resolution of MS/MS was set to 30,000, the The MS/MS resolution was set to 30000, the automatic gain control was set to 1e5, and the maximum accumulation time of ions was set to 80 ms; the dynamic exclusion time was set to 30 s.

8. Determining the False Discovery Rate (FDR) of a Spectrum Match

Percolator was used to determin the False Discovery Rate (FDR) of a Spectrum Match.

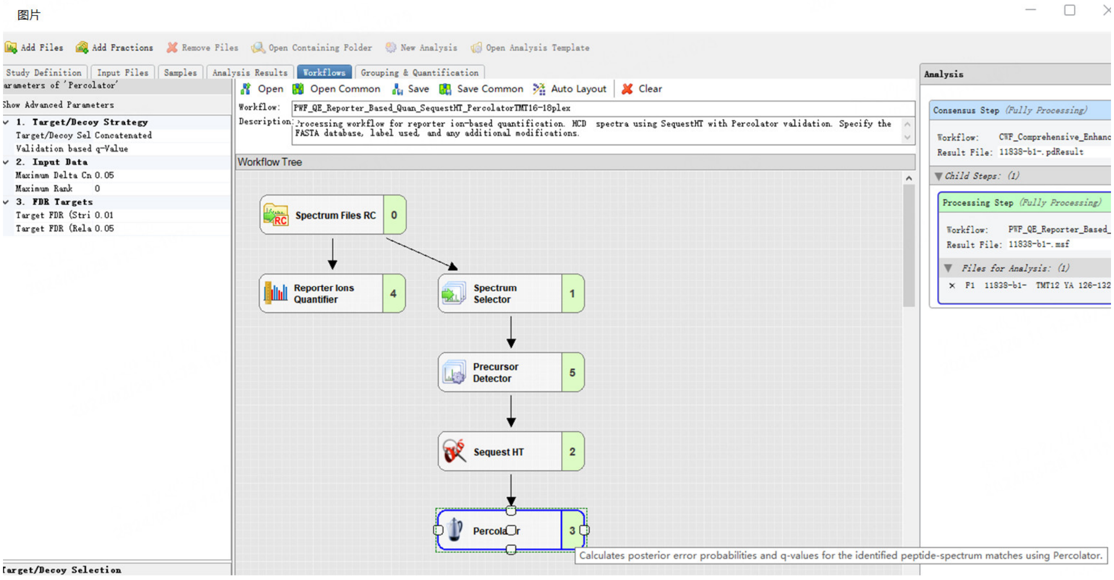

9. Database search

Table.Mass Spectrometry Search Parameters

Items	Settings
Static modification	TMT (N-term, K);Carbamidomethyl(C)
Dynamic modification	Oxidation(M),Acetyl(N-term)
Digestion	Trypsin
Instrument	Orbitrap Fusion
MS1 tolerance	10ppm
MS2 tolerance	0.02Da
Missed Cleavages	2
Database	uniprot-reviewed_yes+taxonomy_10090.fasta
