# Supplementary material for: Integrating Proteomics and Transcriptomics Reveals the Potential Pathways of Hippocampal Neuron Apoptosis in Dravet Syndrome Model Mice
Source: Int J Mol Sci. 2024 Apr 18;25(8):4457. doi: 10.3390/ijms25084457 (PMC11050081; doi:10.3390/ijms25084457)
Supplement: Supplementary file 1 [file ijms-25-04457-s001.zip › supplementary Figures.pdf]

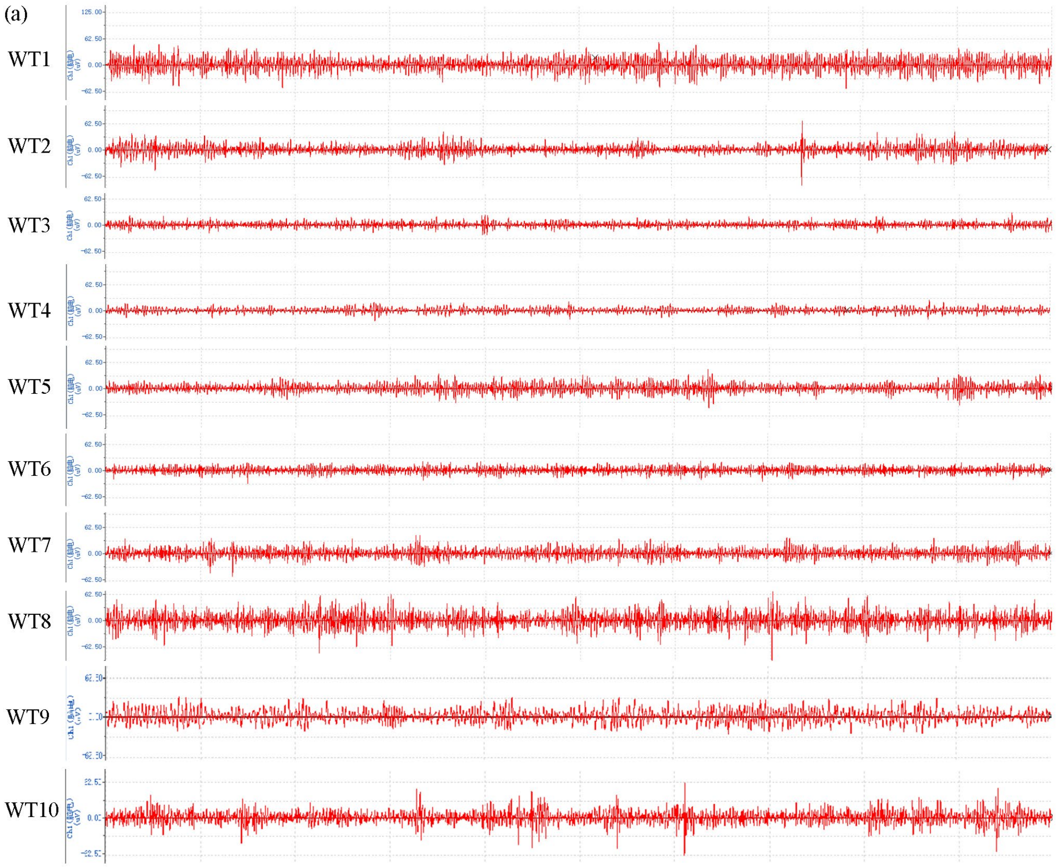
(a)
WT1
WT2
WT3
WT4
WT5
WT6
WT7
WT8
WT9
WT10

(b)

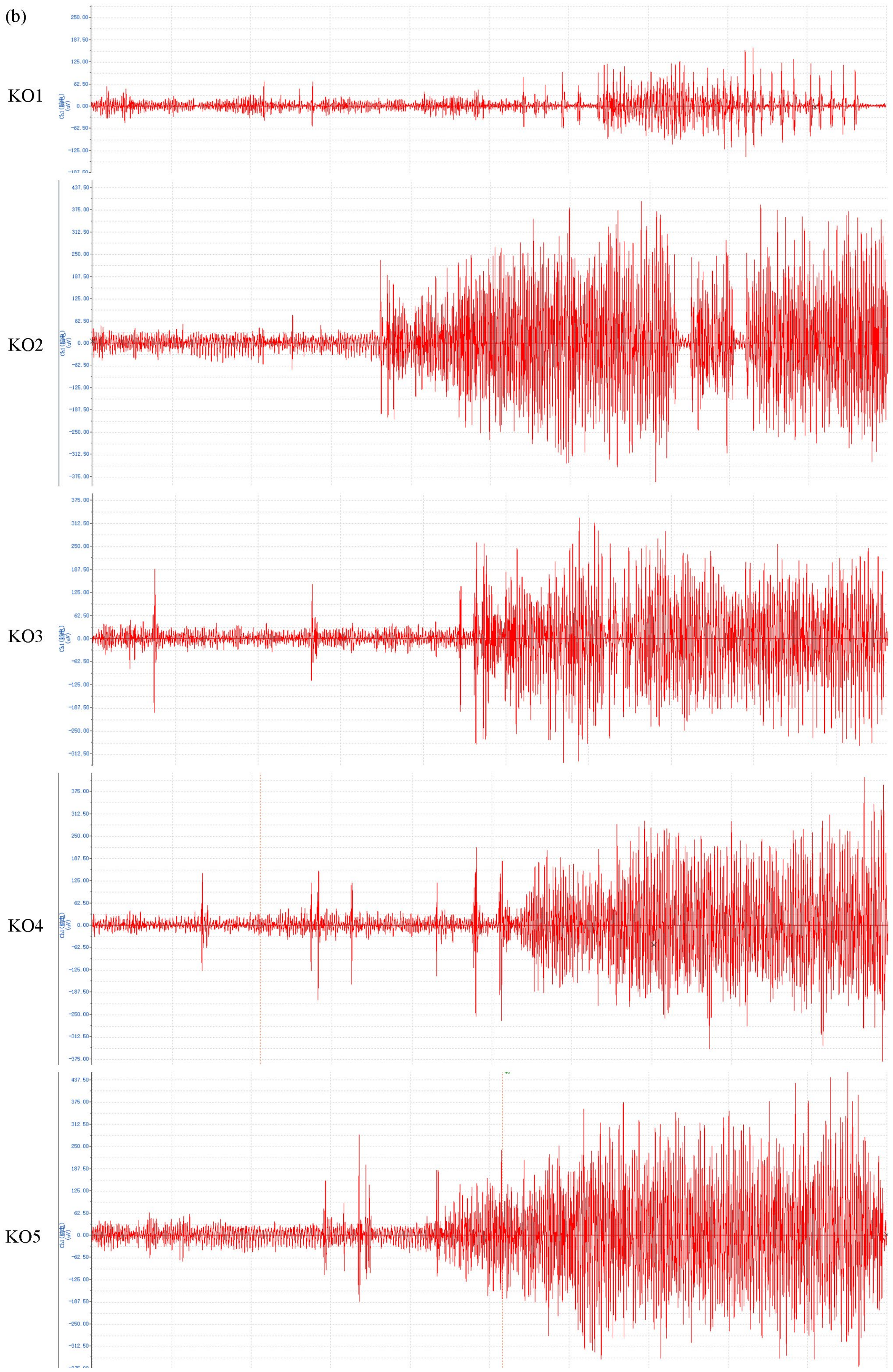

(c)

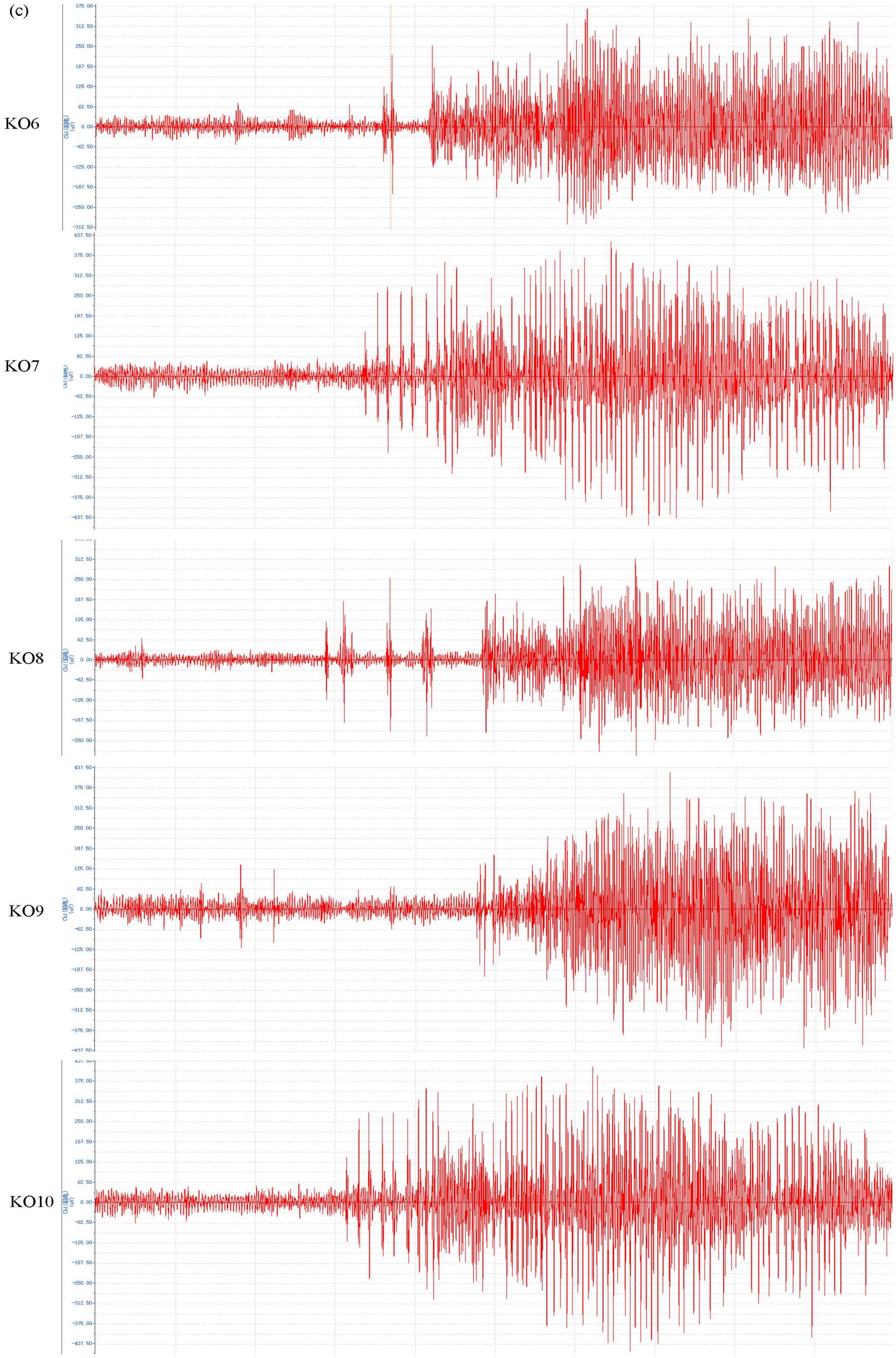

Figure S1.EEG recordings of 10 KO mice and 10 WT mice

(**a**)EEG recordings of WT mice. (**b**)EEG recordings of KO mice number 1to 5. (**c**)EEG recordings of KO mice number 6 to 10.

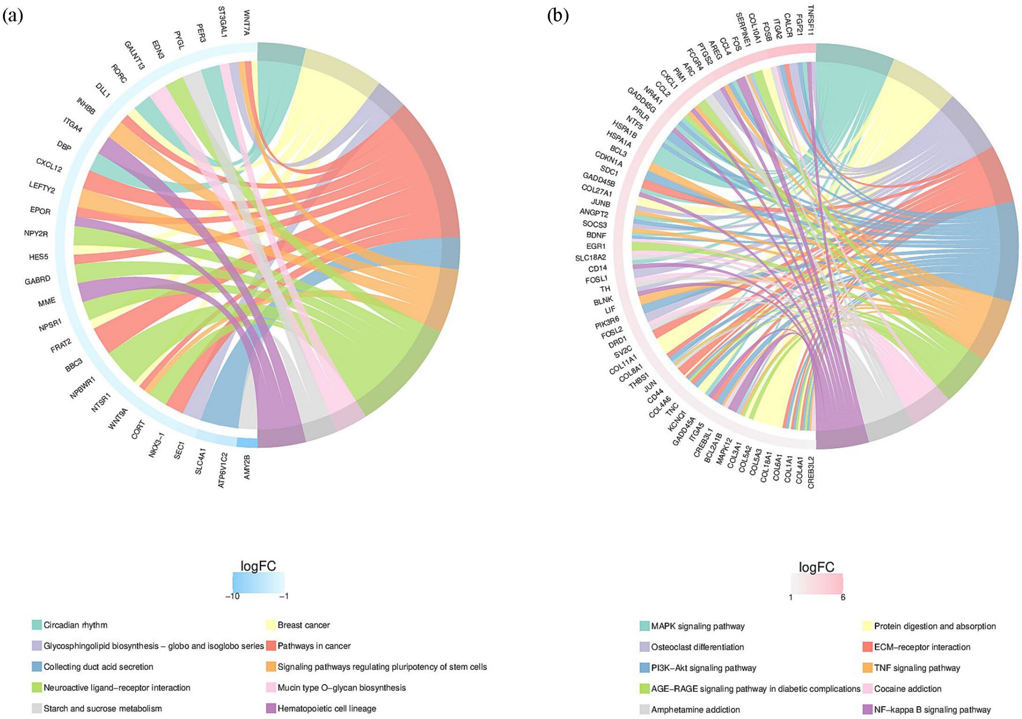

Figure S2.KEGG chord diagram

(**a**) KEGG enriched Top10 chords downregulated differential genes. (**b**)KEGG enriched Top10 chords upregulated differential genes.
